# How do nurse managers describe clinical nurses' work arrangements? A qualitative study

**DOI:** 10.1002/nop2.374

**Published:** 2019-11-13

**Authors:** Ivan Gan

**Affiliations:** ^1^ Department of Arts & Communication University of Houston‐Downtown Houston TX USA

**Keywords:** nurse managers, nurses, staffing, work arrangements

## Abstract

**Aim:**

The researcher sought to understand how nurse managers describe nurses in alternative work arrangements.

**Design:**

The researcher conducted this study using grounded theory.

**Method:**

Semi‐structured interviews. A theoretical sample of 26 baccalaureate‐prepared nurse managers located across the United States participated in the study.

**Results:**

A typology of five work arrangements provides descriptors that contribute toward greater conceptual clarity on nurses' work arrangements. The data reveal that the typology is fluid because nurses can easily switch across work arrangements. Because the rise in alternative work arrangements means that nurses can leave permanent positions—or explore different work arrangements—when circumstances permit or necessitate, nurses who do not receive continued mentoring will likely bring their deficiencies in skill and/or knowledge to facilities where they find future employment. Hence, inadequate mentoring at the unit level has practical consequences for the quality of patient care at the institutional level.

## 1 STUDY

Nurse managers are increasingly using *alternative work arrangements* to achieve adequate staffing amid census fluctuation and nursing shortage (Clendon & Walker, 2016; Gantz et al., 2012; Graham et al., 2014; Kortbeek, Braaksma, Burger, Bakker, & Boucherie, 2015; Kronos, 2017; Larson, Sendelbach, Missal, Fliss, & Gaillard, 2012). Organizational scholars define alternative work arrangements as shift work, temporary work and ‘nonstandard’ employment relations that supplement the ‘standard’ work arrangement of full‐time permanent jobs (Kalleberg, 2000; Katz & Krueger, 2016; Mas & Pallais, 2016; Spreitzer, Cameron, & Garrett, 2017; Van Breugel, Van Olffen, & Olie, 2005). Although alternative work arrangements are neither new to the nursing profession nor to the nursing literature, nursing scholars tend to focus their work on alternative work arrangements in relation to issues such as cost and health outcomes (Maenhout & Vanhoucke, 2013; Xue, Chappel, Freund, Aiken, & Noyes, 2015). This focus exposes a significant knowledge gap in the nuances of nursing shift work (Dall’Ora, Ball, Recio‐Saucedo, & Griffiths, 2016; Harris, Sims, Parr, & Davies, 2015; Rodwell & Fernando, 2016), particularly on nurses' motivations for choosing different work arrangements and the practical consequences for nurse management. Hence, this article offers a typology to explain this organizational phenomenon in the nursing profession and concludes with an applied recommendation for nurse managers.

## 2 BACKGROUND

Adjustments to work schedules are a concern for major stakeholders—facilities, nurses and patients—because scheduling affects cost containment, work‐life balance and the quality of care delivery (Kossek, Rosokha, & Leana, 2019; Thériault, Dubois, Borgès da Silva, & Prud’homme, 2019). Indeed, staffing issues are stressors for nurse managers globally (Fast & Rankin, 2018; Gantz et al., 2012). As patient acuity and census change, nurses on various work arrangements help units maintain nurse‐to‐patient ratios. Nurses who work alternative work arrangements give nurse managers staffing options that they could schedule in advance or on short notice. Without such options, nurse managers oftentimes must multitask between supervisory and clinical roles when their units are direly understaffed (Kossek et al., 2019); the prioritization of patient care is necessary yet it can also draw nurse managers away from their managerial tasks. Although such real‐time staffing changes are a common phenomenon, scholars note the paucity of research on the employment of temporary nurses and the implications of nursing shift work (Dall’Ora et al., 2016; Harris et al., 2015; Rodwell & Fernando, 2016; Simpson & Simpson, 2019). This knowledge gap limits scholars' capacity to discuss practical managerial consequences of the rise in alternative work arrangements. Because alternative work arrangements reshape employment relations in the nursing profession (Baumann, Hunsberger, & Crea‐Arsenio, 2013) and have the potential to affect how nurse managers work, the following research question guided this research study:

How do nurse managers describe nurses in different work arrangements?

## 3 Method

### 3.1 Design

The relative lack of knowledge on nurse managers' work experience in general (Paliadelis & Cruickshank, 2008) and their description of their nurses in varied work arrangements in particular warranted an exploratory study. The researcher used semi‐structured interviews because ‘individuals have unique and important knowledge about the social world that is ascertainable and that can be shared through verbal communication’ (Hesse‐Biber & Leavy, 2011, p. 94). The consolidated criteria for reporting qualitative research and standards for reporting qualitative research guided this research study (O’Brien, Harris, Beckman, Reed, & Cook, 2014; Tong, Sainsbury, & Craig, 2007).

### 3.2 Reflexivity

The researcher conducted the interviews between June 2016–November 2017 as an advanced doctoral student. Prior to conducting this research, the researcher received advanced methodological training in field methods and interview techniques. Other credentials that added depth to his approach to the research design and data collection include professional experience as a former combat medic as well as his present research interest in contemporary career sensemaking. Self‐reflexivity is an important ethical and epistemological consideration because the researcher's personal assumptions provide a lens through which he interprets his participants' lived experiences. As a result, researchers' participation in qualitative research yields different socially constructed meanings as they make sense of their participants' notions of reality (Hesse‐Biber & Leavy, 2011). This coconstruction of subjective realities is a sensemaking process that builds on the researcher's ontological view of multiple versions of ‘truths’ as experienced by the participants.

### 3.3 Data collection

In accordance with the approved institutional review board procedure, colleagues at nurses' professional organizations in the United States disseminated the call for research participants to their members. The researcher did not have a prior relationship with the participants before the interviews, although they knew of his interest in the research topic. The participants were recruited through theoretical sampling until theoretical saturation, where additional participants no longer contribute new information to the conceptual categories (Glaser & Strauss, 1967). The participants scheduled their interviews based on their availability. One‐time, one‐on‐one interviews took place over the telephone without the presence of other individuals. Participants gave written informed consent, and they understood how the researcher would use the data and protect participants' identities. The researcher used the approved interview guide and audio recorded all but one of the interviews. Audio recordings were transcribed verbatim. He took detailed notes throughout and immediately after the interviews.

This study had a sample of 26 participants and an unknown non‐participation rate because the researcher did not have access to the number of potential participants who received the official recruitment statement and those who heard about the study through word‐of‐mouth recruitment. The inclusion criteria required that participants must be registered nurses with clinical management experience and had directly supervised clinical nurses. Participants had an average of 25 years of experience as registered nurses and had on average almost 11 years of experience as nurse managers; their educational background was at least a Bachelor of Science in Nursing. The interviews averaged 47 minutes each. The participants in this article are referred to by assigned pseudonyms.

### 3.4 Data analysis and data trustworthiness

The researcher employed the constant‐comparative method to inductively analyse the data throughout the data collection phase (Strauss & Corbin, 1990). He read the transcript line‐by‐line and used *open coding* to identify concepts of similar meanings. Subsequently, the researcher conducted *axial coding* to develop specific open‐coded categories, which came from grouped categories of initial concepts, based on emerging relationships among the concepts. S*elective coding* transpired through iterative readings of the interview transcript and revisions to the categories, which finally yielded the themes (Strauss & Corbin, 1990). To enhance data trustworthiness, the researcher: (a) discussed emerging patterns in the data with fellow field researchers (Creswell & Miller, 2000); (b) used member checks to verify emerging themes (Miles & Huberman, 1984); (c) used quotations that provide thick, rich descriptions (Creswell & Miller, 2000); and (d) exercised researcher reflexivity.

## 4 RESULTS

One of the first questions on the interview guide was to help the researcher learn what *temporary nurses* meant to the participants. They collectively described temporary nurses as *nurses who 'are not part of our core team'* (Elizabeth) *'but they come in to fill holes'* (Emma). Lily, a paediatric oncology nurse manager with 36 years of registered nurse experience, put it succinctly, ‘Having travellers go in while you're trying to look for permanent employees [is a strategy that] helps your permanent staff’. Participants' definition mirrored the increasing prevalence of team‐based care observed in international healthcare systems (Norful, Martsolf, de Jacq, & Poghosyan, 2017). The similar ways that the participants categorized their nurses in terms of teams created a permanent‐temporary distinction by referencing to their units' staff nurses, nurses' tenure and anticipated tenure on their units. Intuitively, participants viewed temporary nurses as non‐staff nurses.

The permanent–temporary distinction became most salient when participants talked about their expectations of temporary nurses. Charlotte, who has 22 years of nurse manager experience and works at a 500+‐bed teaching hospital, had this expectation, ‘They need to view their role as being part of *a team*, whether it's for a day or for a year’. Michelle, a Millennial in her second year as a nurse manager, narrated her recollection of a travel/agency nurse when she was a nurse assistant:

We had an agency nurse who worked in our unit for a long time. Her contract was up and they renewed it so she was there. Everyone respected her. *She really became part of the team* because she was there for several months and that's how I knew she was an agency nurse.

Productive temporary nurses must not only want to ‘be a *part of the team’* (Emma; Erin), but must also onboard quickly to become ‘*part of the team’* (Heather). Thus, the participants instinctively classified temporary nurses as those who *do not originally belong to their units and whose purpose is to serve immediate needs.*

Participants acknowledged that ‘to bring everyone together *as a team* to work is a challenge’ (Erin). One way that nurse managers onboard temporary nurses was to treat and communicate with them like their staff nurses:

We have huddles every shift so we bring them in where we can introduce them to all the people *on their team* that are coming on right then. And then give them assignments and so that they can all work out there *as a team*. And we include them, you know, wearing an emergency department jacket is a feather in their caps. They get things like that to be *part of the team*. (Emma)

The quotations considered so far reveal that nurse managers view patient care delivery as a collective team effort. Nurse managers and their nurses work toward that goal when staff nurses welcome the help that temporary nurses bring to their units while temporary nurses adapt quickly and perform tasks diligently.

Since permanent/staff nurses and temporary/non‐staff nurses support one another in different ways, this insight suggests that different work arrangements have different features. The next section illustrates the representative features of different work arrangements that make them uniquely attractive to nurses.

### 4.1 Distinguishing different types of temporary and permanent work arrangements

The second part of the analysis builds on the permanent–temporary distinction and presents Table 1 as a visual summary of the distinction between different types of permanent and temporary nurses as well as their similarities and differences. This typology highlights two considerations that the participants used to differentiate among different types of work arrangements. The vertical axis represents a continuum of shorter‐term nurses (who may work a complete shift or a part of it) to longer‐term nurses (whom facilities have scheduled to work for a specified duration or on a permanent basis). The horizontal axis represents a permanent–temporary continuum. Although per diem and travel nurses are more flexible than float and staff nurses are, staff nurses may occasionally float to understaffed units, thereby making staff nurses ‘temporary’ in those instances. Float nurses occupy the centre of this framework because one may view float nurses as permanent (from the perspective of float pool units) yet temporary (from the perspective of the units that use float nurses).

**Table 1:**
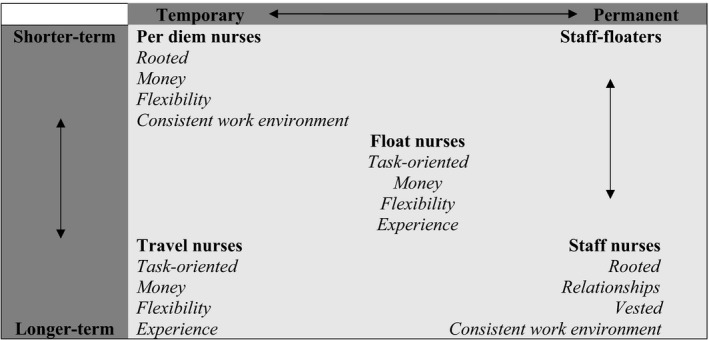
Typology of work arrangements as perceived by nurse managers

The data suggest five major work arrangements—staff nurses, staff‐floaters and three distinct alternative work arrangements: (a) per diem nurses, (b) travel nurses and (c) float nurses. The participants' descriptions of alternative work arrangements aligned with broad categories of temporary nurses reported in the literature (Hemann & Davidson, 2012).

Participants consistently described float, per diem and travel nurses using the descriptors *Money* and *Flexibility*. Other practitioners and researchers have also identified these two descriptors in their work on alternative work arrangements (Adams, Kaplow, Dominy, & Stroud, 2015; Shinners, Alejandro, Frigillana, Desmond, & LaVigne, 2016; Simpson & Simpson, 2019). *Money* refers to differential allowances given to nurses in alternative work arrangements but not to permanent or staff nurses. *Flexibility* refers to both employment flexibility (i.e., the many configurations of work schedules in which facilities can employ nurses) and nurses' flexibility (to adapt to the challenges they find at work). Illustrative quotations for these five work arrangements will be provided below.

#### 4.1.1 Staff nurses

##### Rooted

Like per diem nurses, staff nurses opt for a work arrangement that keeps them in their communities. Although nurses know that they can earn differential allowances by picking up additional shifts as per diem nurses or by signing contracts as travel nurses, Ella (who works on a 42‐bed post‐operative joint‐replacement unit) said that some nurses preferred permanent positions chiefly because family responsibilities kept them rooted in their local areas. Sophia, who works at a 900+‐bed non‐profit acute care hospital, shared her analysis with me, ‘Travellers are the ones who don't have family obligations at home because it's very difficult when you've children …Usually, travelling's seen as an early job entry or work after kids are grown’. Emma, employed at a large level‐1 trauma tertiary care centre, recognized the same reasons and disclosed the curiosity that almost every nurse whom she had met had about the adventures of travel nursing:

[Permanent nurses] don't have the ability to go anywhere. They've kids in school. They don't want to be out there travelling. They've home obligations. We've lost a fair number of nurses [who became travel nurses]. Some are older. Some of the ones who've left are younger. …sometimes you can't blame them for why they go out to see what's out there.

Indeed, travel nurses may talk about what ‘it was like someplace else. The nurse that's just in this hospital all the time sometimes gets fascinated by all the stories they [travel nurses] have to tell’ (Abigail). Mia, who supervises other nurse managers at a 200+‐bed community hospital, concurred, ‘Most of them have family connections or whatever that keeps them here. Most travel nurses, while they'll share their stories and the glamor of travelling all over the world, don't try to influence the nurses here to go travel’. It seems that staff nurses will try travel nursing if they can but they value their local relationships most and therefore they prefer permanent positions. Mia's account suggests that the employment of temporary nurses can affect existing permanent nurses (Simpson & Simpson, 2019), who might rethink about their permanent/traditional work arrangements.

Participants' view of *Rooted* staff nurses appears negatively framed, as they perceive that staff nurses will travel or work elsewhere if they do not have family obligations. Staff nurses may also shoulder significant financial burden, which requires them to work permanently in consistent full‐time staff positions as opposed to flexible arrangements or shorter hours.

##### Relationships

Nurse managers viewed staff nurses as not only valuing familial relationships but also collegial relationships. Three participants identified their staff nurses as ‘my people’, suggesting a distinction between permanent nurses and temporary nurses. Kimberly (a third‐year nurse manager with 20 years of registered nurse experience) established a work culture based on trust and open communication:

I'm a little more transparent with the people who belong to me. Because they're mine and they understand that's the way I'm. I'm a big believer that you should be very transparent in all aspects of the organization–fiscal, regulatory–everything that I know, my people know.

Participants built relationships through open communication with their staff nurses, especially with increased proximity and interactional frequency. Olivia, who works at a 1,000+‐bed teaching hospital and has eight years of nurse manager experience, discerned these relational differences:

You tend to know your permanent employees better. A permanent employee is more likely to tell you things than the temporary [employee]. The temporary people don't know you; they're only there for a short period of time, so they might not be as forthcoming. You also don't know their background as well, you don't know their personalities as well.

Nevertheless, Ashley said, ‘When they wanted to join us, it was because they recognized that the teaming and the culture we had on our units was something that they wanted to be a part of’. The right work culture keeps nurses and cultivates relationships.

The relationships that nurse managers foster with their staff nurses have a greater social and personable element, in contrast to the more transactional approach usually observed with temporary workers (Van Breugel et al., 2005). Nurse managers relate to their staff nurses better because they view staff nurses as integral members of their teams’ core.

##### Vested

Because of their rootedness and relationships, staff nurses have a stake in their organizations and they voluntarily articulate ways that their units can change or improve. Lily explained:

They sometimes raise more things that have to do with the unit and the team and they look at issues in the unit… because they're going to be there for the long haul, if there's something that's frustrating in the workflow… they're more engaged at trying to improve those things, whereas the traveller knows that he or she will be gone in 13 weeks.

Staff nurses' commitment reveals their high member identification with their organizations (Van Breugel et al., 2005). They communicate their thoughts more frequently because they take ownership of the concerns that they have—or problems that they observe—in their work environments. If they remain silent, they must then accept the consequences and the work conditions. This descriptor indicates that the anticipated permanence of one's job will make the employee more vocal about improvements as compared to more itinerant employees and those with less frequent or shorter footholds.

##### Consistent work environment

Finally, staff nurses find comfort in familiarity. Abigail categorized them this way:

They don't like to get outside of their box. They're very comfortable in their geographical location, they've four walls, they know the patient population that they're dealing with, they know their coworkers, they know where their stuff is, they know predictably what's going to happen on any given day; they may have a busy day or a slow day, but the days are pretty much the same and that comfort level is good for some people. Some people like that, some people need that.

This final descriptor ties together the preceding descriptors and provides an overarching explanation as to why permanent nurses stay as permanent nurses, despite the attractive features of other work arrangements.

#### 4.1.2 Staff‐floaters

Staff‐floaters are a subset of staff nurses who are functionally temporary nurses who fill gaps in understaffed units (top‐right quadrant of Table 1). However, this is not an alternative work arrangement because these nurses become contingent temporary nurses due to organizational necessity and not necessarily by their choice. Hospitals may float their staff nurses when required, said Sarah, ‘At our facility you've to float. If it's within your scope, you really don't have a choice. It follows a rotation in each department. … [An] overstaffed floor will send a nurse to the floor needing help’. Amelia disclosed, ‘I'm a working manager, meaning that I'll go along and work beside them. So, if we're short‐staffed, I'll be that float person to go in and work with that team through the day’. Indeed, nurse managers are typically one of the most senior registered nurses on their units who also deliver nursing care and who serve as a resource to their nurses (Paliadelis & Cruickshank, 2008). Hence, staff‐floaters are characteristically staff nurses and they retain their staff nurse descriptors because their main responsibilities are still with their primary units.

#### 4.1.3 Per diem nurses

##### Rooted

These nurses live in the local area and work part‐time mostly for work‐life balance. Elizabeth (18th‐year nurse manager with 31 years of registered nurse experience) shared with me a conversation she recently had with a per diem nurse:

She's so happy doing that. … That position lends itself to even more control over her schedule. … She wants to have the perfect work‐life balance. She doesn't want to work any Tuesdays because that's when her sons have soccer. She doesn't want to work any Fridays because that's the day she takes yoga class. And so she works per diem [so that] she can completely control when she works …and she gets paid a tiny bit more to do that. So, I think there's a financial incentive as well as complete control over her schedule.

Michelle also gave a similar account based on her experience with temporary nurses in general and per diem nurses in particular, ‘Their husbands are working so they're kind of bringing in extra income but they don't have to work full‐time and they need the flexibility to work around child care’. Per diem nurses cherish flexibility because they prioritize their non‐work lives; they prefer to dedicate their energies to family responsibilities and to participate in personal activities meaningful to them.

Nurse managers construed per diem nurses' rootedness more positively than how they characterized staff nurses' rootedness. They viewed per diem nursing as an autonomous choice, implying that per diem nurses ‘work to live’ as they valued engagements outside of work while staff nurses ‘live to work’ because of family and financial obligations. Participants' perception of per diem nurses chiefly reflected young mothers who chose not to work full‐time because of active exercise regimens and childrearing duties. This descriptor alludes to the value that these nurses place on their communal relationships, such as those with their nuclear families, and non‐work engagements at their local gyms. Thus, participants perceived per diem nurses as those who embrace work‐life balance, who do not shoulder their families' main financial burden and who view the extra income as an added benefit.

##### Money

Aligned with the analysis for the earlier descriptor, participants perceived per diem nursing as an alternative source of supplemental income. Erin, who works at a Veterans Affairs hospital, commented, ‘They're there for the money, to make extra money. They're there, they're out’. Just as in Michelle's comments in the earlier descriptor, some of the per diem nurses work to supplement their families’ main income source.

##### Flexibility

Elizabeth gave a hypothetical example where per diem nurses at her hospital could choose their schedules, ‘You can call Sue and say, “Hey, can you pick up a shift tomorrow? We're really busy.” And then she can say yes or no as long as she's already met her other monthly requirements’. This descriptor ties back to *Money* and that these nurses can pick up last‐minute shifts because their *Rooted* presence in the local community makes proximity less of an issue.

##### Consistent work environment

They not only get to choose when they work but also where they work. Tiffany, who works on an intensive care unit at a level‐1 trauma teaching hospital, analysed, ‘PRNs usually tend to fall into two different groups. There're nurses that only work for us and they're PRN for us, or they're nurses who've another full‐time job and they'll work PRN for us’. Michelle also gave an example, ‘I've one who's a nurse practitioner who wants to stay on in our unit after she has finished nurse practitioner school’. Nurses may also view per diem nursing as a way ‘to get their foot in the door’, as Erin told me about a nurse who worked a once‐a‐week schedule for her even though this nurse had a full‐time position at a hospital closer to where she lived.

Per diem nurses commonly want a consistent work environment because they value familiarity with their employers, colleagues and community. They also work per diem so that they increase their access to future employment opportunities.

#### 4.1.4 Travel nurses

##### Task‐oriented

Participants complimented travel nurses as competent and independent. However, they noticed that travel nurses had less interest in cultivating relationships with patients than nurses in other work arrangements. Stephanie, who has 40 direct reports and works in a neurosurgery department at a level‐1 trauma hospital, remarked, ‘They're not very concerned about individual care of patients. They do the orders. They follow the orders but …they know they're just there a short time. They're not very attached to the patients at all’. This criticism seems largely consistent among other participants. Lily praised travellers as ‘relatively good nurses’ but they only ‘do their job on the unit’ and not engage with others as much as permanent nurses. Abigail had an explanation as to why travellers kept mostly to themselves, ‘Some of them are very gregarious but some of them are rather quiet, and they may have been burned somewhere else, and so they sort of stay to themselves until they know that they're welcomed’. It seems plausible that travel nurses focus mostly on completing tasks because they fill staffing gaps at their temporary units.

##### Money

Because travel nurses possess sought after expertise, they seek employment opportunities that will pay them well. Charlotte works for a hospital network in an urban but rather isolated Northeastern region that has difficulty retaining nurses:

We don't typically get local nurses, we get nurses that like to travel home for a week at a time. … They want to go back home and to their families, which we completely understand …they're really doing it financially, because maybe there aren't local opportunities, or the salaries locally are so low. These are primary breadwinners.

Similarly, Olivia qualified the perception that travellers ‘make a lot more money, because they're willing to go at a moment's notice’. However, to earn that higher remuneration, they must have the tenacity to adapt quickly to different geographical and work environments.

##### Flexibility

Olivia stated, ‘They tend to be very well‐trained, they can jump right in’, meaning that travel nurses do not get the lengthier orientation that new graduate nurses typically receive. Evelyn, a nurse manager at an orthopaedic hospital, agreed, ‘They're expected to adapt really quickly to their roles. They don't need a lot of orientation. They've learned how to get you through a rough patch in your staffing’. Much of their flexibility comes from having gained exposure to and experience in different settings.

##### Experience

They travel and work as a lifestyle choice. Lily outlined two types of travel nurses, ‘The two groups of travellers—there's the one that travels for money and the one that travels so that they can travel. They go to places that they wouldn't ordinarily be able to go to’. This descriptor shares similarities with the same namesake for float nurses: travel nurses thrive in geographically dispersed locations while float nurses thrive in different units in their organizations.

#### 4.1.5 Float nurses

##### Task‐oriented

Participants generally had a positive impression of float nurses' competency, but they also remarked that float nurses seemed less interested in patient care than nurses in other work arrangements other than travel nurses. Stephanie, recognizing float nurses' permanent employment status and affiliation with their hospitals, concluded, ‘They are a little more involved with taking care of their patients, so a little bit more involved with knowing the staff and speaking to the staff. Travel nurses pretty much come in, do their job and leave’. Mia shared the same view. She opined that float nurses had higher levels of ownership than travel nurses and would strive ‘to interact very positively with the patients no matter [which] unit they're working on’.

##### Money

Stephanie estimated, ‘Travellers have probably the highest rate of pay and the temporary float pool nurses have the next highest rate of pay’. All three alternative work arrangements share this descriptor. Staff‐floaters do not share this descriptor because they are essentially staff nurses who float (and become temporary nurses temporarily) not by their choice but due to fluctuating patient acuity and census. This descriptor explains that nurses who voluntarily work alternative work arrangements receive differential allowances in recognition of their versatile clinical skills as well as their willingness to work in different clinical settings and teams on very little notice. Expressed simply, hospitals reward nurses who are willing to fill urgent staffing gaps.

##### Flexibility

They provide their organizations with added staffing flexibility, ‘Float nurses came up pretty much through shortage and through seeing agencies out there doing this. It was a way of mimicking the agencies’ (Avery). Nevertheless, Jessica (progressive care unit, level‐1 trauma) observed that float nurses must adapt quickly to new environments, ‘When you're a float nurse, you should have the ability to step into any situation, orient quickly and be able to take over the job’. This descriptor connects back to the earlier descriptor because differential allowances partly compensate nurses for their flexibility in meeting units’ pressing needs.

##### Experience

Jessica continued, ‘They like the excitement of going from place to place. They enjoy the mix of patients: taking care of a transplant patient one day, a neuro patient another day and a cardiology patient another day’. Float nurses look forward to the daily challenge of working with a different patient mix and applying different skills. They also get to experience novel settings and in this sense are adventurous like travel nurses: they get to explore different clinical specialties in their hospitals without travelling afar.

## 5 DISCUSSION

This study unearths nurse managers' conception of the permanent–temporary distinction of nurses. Participants' definition of temporary nurses offers three fundamental insights into how they view their nurses. First, nurse managers view permanent nurses as those who work exclusively/mostly for their units; they express a degree of belongingness of permanent nurses as members who provide dependable and long‐term stability to their respective units. By implication, individuals share common work identities (Van Breugel et al., 2005), appreciate one another's personalities and foster workplace relationships developed over time and into the future as they anticipate continued interactions as colleagues on the same unit.

Second, nurse managers shoulder the responsibility of finding replacement nurses—whether temporary or permanent—for their understaffed teams. The second insight reinforces the previous insight regarding the seriousness of nursing work. It also underscores key managerial tasks of maintaining nurse‐to‐patient ratios and human resource functions such as recruitment and retention (Kortbeek et al., 2015; Larson et al., 2012; Thériault et al., 2019). A wise reminder from Kimberly:

[What] I don't always remember is that the people are first. We always get caught up in the processes and in the payments, but it's all about the people. And if I can remember that, my team will do very well.

The two‐dimensional framework depicts nurse managers' categorization and perception of nurses' work arrangements. Participants expect temporary (or non‐permanent) nurses to be highly competent and that they will ‘hit the ground running’ (Amanda; Avery; Isabella; Lily). Because of their clinical proficiency and flexibility, nurses in alternative work arrangements typically receive differential allowances (and, therefore, higher salaries) than staff nurses. Highly productive temporary nurses ‘blend in with the rest of the staff’ (Abigail) and help units meet short‐term needs. From another perspective, alternative work arrangements give units that do not wish to—or cannot—invest in the mentoring of new graduate nurses an alternative source of experienced nurses.

Third, an explication of how nurse managers describe their nurses' work arrangements reveals a key presupposition: nurse managers think of their nurses in terms of teams. Participants and their staff nurses expect temporary nurses to adapt expeditiously and contribute as part of the team. They typically provide temporary nurses with an abbreviated orientation to get them off ‘on the right foot’ (Emily) and ‘up to speed’ (Abigail; Emily; Michelle; Olivia). The emphasis on getting temporary nurses ‘up and running’ (Abigail; Sophia) ‘right out of the gate’ (Harper) underscores that time is of the essence, therefore acknowledging the use of alternative work arrangements as a clinical approach toward meeting patient care standards as well as a strategic organizational feature of human resource management.

Participants are concerned about whether temporary nurses' personalities will fit in with their teams. Just as they instinctively perceive the permanent–temporary distinction from their units' perspective, nurse managers are concerned about how their staff nurses will receive temporary nurses because staff nurses ‘usually raise issues about teaming’ (Ashley). Ashley summarized that the temporary nurses whom her staff nurses enjoyed working with were ‘so good at being nurses they can just jump in and be a regular *part of the team’.* Harper (non‐profit hospital, level‐2 trauma) stated similarly:

If within their [temporary nurses'] personality they buy‐in to the type of care that we wish to have for our patients, we don't have an issue with them. If they're doing their best, if they're competent and they buy‐in to *the team concept* of being helpful to other nurses in reciprocal as being helpful, we don't have a problem. If we've nurses who don't have the personality, they're just coming in there for maybe the money and doing the job and they're not wholeheartedly bought‐in into the best patient care, it's just a job to them, then we've an issue.

Therefore, whenever possible, nurse managers interview temporary nurses carefully to ascertain candidates' personalities and they assign incoming nurses (when within their control and when circumstances permit) to work with nurses whom they envision will work well together.

### 5.1 An applied recommendation

This study's analysis offers a framework to understand how nurse managers describe nurses' work arrangements. The data reveal that the typology is fluid because nurses can switch among work arrangements to achieve what they desire from working as nurses. That insight empirically supports the notion that work arrangements can reflect work motivations, which may influence whom and how nurse managers mentor (Gan, 2019a). Nurse managers invest much of their time and resources in mentoring nurses (Kodama & Fukahori, 2017; Sveinsdóttir, Ragnarsdóttir, & Blöndal, 2016). Mentoring helps to disseminate knowledge and to promote evidence‐based practice (Abdullah et al., 2014; Karlberg Traav, Forsman, Eriksson, & Cronqvist, 2018) as well as to impart career insights and to foster supportive relationships (Simpson & Simpson, 2019). Although it is intuitive to recommend that nurse managers should strategically allocate limited mentoring resources to their permanent nurses, the rise in alternative work arrangements means that nurses can leave permanent positions—or explore different work arrangements—when circumstances permit or necessitate. Because staffing is increasingly transient, nurse managers should consider mentoring every nurse—regardless of work arrangement—when opportunities arise. Although this recommendation will burden nurse managers further in the short‐term, it has the potential to create a long‐term culture that organically geminates mentoring relationships to alleviate some of future nurse managers' stress with mentoring and scheduling, as the nursing literature notes that mentors and mentees benefit professionally from mentoring relationships (Hale, 2018; McBride, Campbell, & Deming, 2019). Given the changing nature of staffing strategies and the varied work arrangements from which nurses can choose, it is important to emphasize that this recommendation is about raising the overall quality of care delivery by addressing inadequate mentoring. Because nurses who do not receive continued mentoring will likely bring their deficiencies in skill and/or knowledge to facilities where they find future employment (Gan, 2019b), inadequate mentoring at the unit level has practical consequences for the quality of patient care at the institutional level.

The above recommendation is distinct from merely stating that the employment of temporary nurses in itself—without considering the quality of the employed temporary nurses—will affect patient care. Indeed, as the employment of temporary nurses to maintain adequate staffing implies staffing instability (Thériault et al., 2019), healthcare administrators are typically concerned about whether increased employment of nurses in alternative work arrangements will adversely affect the quality of patient care (Morelock & Kirk, 2019); however, this instinctive concern is largely unsupported by empirical studies (Simpson & Simpson, 2019). Recent studies indicate that the resources—such as time—that nurses have, nurses' work experience and nurse‐to‐patient ratios affect the extent to which nurses complete patient care tasks such as the monitoring of patients' fluid intake (Litchfield, Magill, & Flint, 2018) and the taking of patients' vital signs (Recio‐Saucedo et al., 2018). The delivery and monitoring of these tasks are important because they are within nurses' scope of practice and they directly affect patients' health outcomes (Stalpers, De Vos, Van Der Linden, Kaljouw, & Schuurmans, 2017). Such nuances in nurses' work, nurses' qualifications and motivations as well as the challenges that nurses confront daily highlight salient factors that facilities must constantly evaluate as they ascertain and manage the staffing mix appropriate for their respective circumstances. These factors are especially crucial as the cost and the quality of patient care can create competing interests, complicating the allocation of mentoring resources.

## 6 CONCLUSION

Alternative work arrangements both complement and challenge the conventional or ‘standard’ boundaries of work and personal time as variations in work arrangements shape nurses' experience of work time and structures such as schedules. Nurse managers who understand the varied motivations undergirding their nurses' preference for particular work arrangements are better equipped to mitigate staffing challenges, manage the potential impact that staffing mix has on patient care and effectively fulfil their mentoring responsibility.

The study's sample is a limitation because staffing demands and licensing requirements differ from state to state and from other international jurisdictions. Participants' broad clinical backgrounds may limit transferability of findings. However, the consistent themes reported by the participants ring true despite their diverse clinical settings.

Future studies should seek greater insights into alternative work arrangements' impact on nurses' careers. As temporary arrangements increase the likelihood of temporary employees developing transactional rather than relational interactions with their employers (Van Breugel et al., 2005), nurses' experience of work time shapes the way they relate to their colleagues and vice versa. For instance, although nursing researchers have studied mentoring extensively and have reported that Millennial nurses in particular want ongoing mentoring and flexible careers (Hale, 2018; Jamieson, Kirk, Wright, & Andrew, 2015), the nursing scholarship sheds limited light on how alternative work arrangements affect mentoring as a communicative behaviour (Gan, 2019a). As Millennial nurses will have increasingly diverse nursing career options (Jamieson et al., 2015), researchers should invest greater effort into understanding Millennial nurses' thoughts about alternative work arrangements.

## ETHICAL APPROVAL

The Texas A&M University IRB approved this study.

## CONFLICT OF INTEREST

No conflict of interest has been declared by the author.

## References

[nop2374-bib-0001] Abdullah, G. , Rossy, D. , Ploeg, J. , Davies, B. , Higuchi, K. , Sikora, L. , & Stacey, D. (2014). Measuring the effectiveness of mentoring as a knowledge translation intervention for implementing empirical evidence: A systematic review. Worldviews on Evidence‐Based Nursing, 11, 284–300. 10.1111/wvn.12060 25252002 PMC4285206

[nop2374-bib-0002] Adams, J. , Kaplow, R. , Dominy, J. , & Stroud, B. (2015). Beyond a Band‐Aid^®^ approach: An internal agency solution to nurse staffing. Nursing Economics, 33, 51. 26214939

[nop2374-bib-0003] Baumann, A. , Hunsberger, M. , & Crea‐Arsenio, M. (2013). Full‐time work for nurses: Employers’ perspectives. Journal of Nursing Management, 21, 359–367. 10.1111/j.1365-2834.2012.01391.x 23410116

[nop2374-bib-0004] Clendon, J. , & Walker, L. (2016). Nurses aged over 50 and their perceptions of flexible working. Journal of Nursing Management, 24, 336–346. 10.1111/jonm.12325 26119711

[nop2374-bib-0005] Creswell, J. W. , & Miller, D. L. (2000). Determining validity in qualitative inquiry. Theory into Practice, 39, 124–130. 10.1207/s15430421tip3903_2

[nop2374-bib-0006] Dall’Ora, C. , Ball, J. , Recio‐Saucedo, A. , & Griffiths, P. (2016). Characteristics of shift work and their impact on employee performance and wellbeing: A literature review. International Journal of Nursing Studies, 57, 12–27. 10.1016/j.ijnurstu.2016.01.007 27045561

[nop2374-bib-0007] Fast, O. , & Rankin, J. (2018). Rationing nurses: Realities, practicalities and nursing leadership theories. Nursing Inquiry, 25(2), e12227 10.1111/nin.12227 29277951

[nop2374-bib-0008] Gan, I. (2019a). Alternative work arrangements: Reshaping the future of nurses’ workplace communication and relationships. Nursing Forum, 54, 227–231. 10.1111/nuf.12321 30566243

[nop2374-bib-0009] Gan, I. (2019b). How do nurses’ work arrangements influence nurse managers’ communication? A qualitative study. Journal of Nursing Management. Advance online publication. 10.1111/jonm.12817 31211906

[nop2374-bib-0010] Gantz, N. R. , Sherman, R. , Jasper, M. , Choo, C. G. , Herrin‐Griffith, D. , & Harris, K. (2012). Global nurse leader perspectives on health systems and workforce challenges. Journal of Nursing Management, 20, 433–443. 10.1111/j.1365-2834.2012.01393.x 22591145

[nop2374-bib-0011] Glaser, B. G. , & Strauss, A. (1967). The discovery of grounded theory: Strategies for qualitative research. Chicago, IL: Aldine.

[nop2374-bib-0012] Graham, E. , Donoghue, J. , Duffield, C. , Griffiths, R. , Bichel‐Findlay, J. , & Dimitrelis, S. (2014). Why do older RNs keep working? JONA: the Journal of Nursing Administration, 44, 591–597. 10.1097/NNA.0000000000000131 25340924

[nop2374-bib-0013] Hale, R. (2018). Conceptualizing the mentoring relationship: An appraisal of evidence. Nursing Forum, 53, 333–338. 10.1111/nuf.12259 29691870

[nop2374-bib-0014] Harris, R. , Sims, S. , Parr, J. , & Davies, N. (2015). Impact of 12h shift patterns in nursing: A scoping review. International Journal of Nursing Studies, 52, 605–634. 10.1016/j.ijnurstu.2014.10.014 25468281

[nop2374-bib-0015] Hemann, M. , & Davidson, G. (2012). Perspective of a float pool model in ambulatory care. MedSurg Nursing, 21, 164–170. 22866437

[nop2374-bib-0016] Hesse‐Biber, S. N. , & Leavy, P. (2011). The practice of qualitative research (2nd ed.). Thousand Oaks, CA: Sage.

[nop2374-bib-0017] Jamieson, I. , Kirk, R. , Wright, S. , & Andrew, C. (2015). Generation Y New Zealand Registered Nurses’ views about nursing work: A survey of motivation and maintenance factors. Nursing Open, 2, 49–61. 10.1002/nop2.16 27708801 PMC5047315

[nop2374-bib-0018] Kalleberg, A. L. (2000). Nonstandard employment relations: Part‐time, temporary and contract work. Annual Review of Sociology, 26, 341–365. 10.1146/annurev.soc.26.1.341

[nop2374-bib-0019] Karlberg Traav, M. , Forsman, H. , Eriksson, M. , & Cronqvist, A. (2018). First line nurse managers’ experiences of opportunities and obstacles to support evidence‐based nursing. Nursing Open, 5, 634–641. 10.1002/nop2.172 30338109 PMC6178359

[nop2374-bib-0020] Katz, L. F. , & Krueger, A. B. (2016). The rise and nature of alternative work arrangements in the United States, 1995–2015 (NBER Working Paper No. 22667). Cambridge, MA: National Bureau of Economic Research.

[nop2374-bib-0021] Kodama, Y. , & Fukahori, H. (2017). Nurse managers’ attributes to promote change in their wards: A qualitative study. Nursing Open, 4, 209–217. 10.1002/nop2.87 29085647 PMC5653397

[nop2374-bib-0022] Kortbeek, N. , Braaksma, A. , Burger, C. A. J. , Bakker, P. J. M. , & Boucherie, R. J. (2015). Flexible nurse staffing based on hourly bed census predictions. International Journal of Production Economics, 161, 167–180. 10.1016/j.ijpe.2014.12.007

[nop2374-bib-0023] Kossek, E. E. , Rosokha, L. M. , & Leana, C. (2019). Work schedule patching in health care: Exploring implementation approaches. Work and Occupations. Advance online publication. 10.1177/0730888419841101 PMC8623715 34840412

[nop2374-bib-0024] Kronos (2017). Nurses and fatigue survey report. Internal Kronos Report, unpublished.

[nop2374-bib-0025] Larson, N. , Sendelbach, S. , Missal, B. , Fliss, J. , & Gaillard, P. (2012). Staffing patterns of scheduled unit staff nurses vs. float pool nurses: A pilot study. MedSurg Nursing, 21, 27–39. 22479872

[nop2374-bib-0026] Litchfield, I. , Magill, L. , & Flint, G. (2018). A qualitative study exploring staff attitudes to maintaining hydration in neurosurgery patients. Nursing Open, 5, 422–430. 10.1002/nop2.154 30062036 PMC6056434

[nop2374-bib-0027] Maenhout, B. , & Vanhoucke, M. (2013). An integrated nurse staffing and scheduling analysis for longer‐term nursing staff allocation problems. Omega, 41, 485–499. 10.1016/j.omega.2012.01.002

[nop2374-bib-0028] Mas, A. , & Pallais, A. (2016). Valuing alternative work arrangements (NBER Working Paper No. 22708). Cambridge, MA: National Bureau of Economic Research.

[nop2374-bib-0029] McBride, A. B. , Campbell, J. , & Deming, K. (2019). Does having been mentored affect subsequent mentoring? Journal of Professional Nursing, 35, 156–161. 10.1016/j.profnurs.2018.11.003 31126390

[nop2374-bib-0030] Miles, M. B. , & Huberman, A. M. (1984). Qualitative data analysis: A sourcebook of new methods (2nd ed.). Thousand Oaks, CA: Sage.

[nop2374-bib-0031] Morelock, S. G. , & Kirk, J. D. (2019). An urban medical system's exploratory study of medication errors. Nursing Open, 6, 1197–1204. 10.1002/nop2.319 31367446 PMC6650646

[nop2374-bib-0032] Norful, A. , Martsolf, G. , de Jacq, K. , & Poghosyan, L. (2017). Utilization of registered nurses in primary care teams: A systematic review. International Journal of Nursing Studies, 74, 15–23. 10.1016/j.ijnurstu.2017.05.013 28595110 PMC5650533

[nop2374-bib-0033] O’Brien, B. C. , Harris, I. B. , Beckman, T. J. , Reed, D. A. , & Cook, D. A. (2014). Standards for reporting qualitative research. Academic Medicine, 89, 1245–1251. 10.1097/acm.0000000000000388 24979285

[nop2374-bib-0034] Paliadelis, P. , & Cruickshank, M. (2008). Using a voice‐centered relational method of data analysis in a feminist study exploring the working world of nursing unit managers. Qualitative Health Research, 18, 1444–1453. 10.1177/1049732308322606 18832771

[nop2374-bib-0035] Recio‐Saucedo, A. , Maruotti, A. , Griffiths, P. , Smith, G. B. , Meredith, P. , Westwood, G. , … Schmidt, P. (2018). Relationships between healthcare staff characteristics and the conduct of vital signs observations at night: Results of a survey and factor analysis. Nursing Open, 5, 621–633. 10.1002/nop2.179 30338108 PMC6177549

[nop2374-bib-0036] Rodwell, J. , & Fernando, J. (2016). Managing work across shifts: Not all shifts are equal. Journal of Nursing Scholarship, 48, 397–405. 10.1111/jnu.12220 27228576

[nop2374-bib-0037] Shinners, J. , Alejandro, J. A. N. , Frigillana, V. , Desmond, J. , & LaVigne, R. (2016). Quality improvement: Creating a float pool specialty within a new graduate residency. MedSurg Nursing, 25, 79–84. 27323464

[nop2374-bib-0038] Simpson, K. , & Simpson, R. (2019). What do we know about our agency nurse population? A scoping review. Nursing Forum. Advance online publication. 10.1111/nuf.12361 31292974

[nop2374-bib-0039] Spreitzer, G. M. , Cameron, L. , & Garrett, L. (2017). Alternative work arrangements: Two images of the new world of work. Annual Review of Organizational Psychology and Organizational Behavior, 4, 473–499. 10.1146/annurev-orgpsych-032516-113332

[nop2374-bib-0040] Stalpers, D. , De Vos, M. L. G. , Van Der Linden, D. , Kaljouw, M. J. , & Schuurmans, M. J. (2017). Barriers and carriers: A multicenter survey of nurses’ barriers and facilitators to monitoring of nurse‐sensitive outcomes in intensive care units. Nursing Open, 4, 149–156. 10.1002/nop2.85 28694979 PMC5500986

[nop2374-bib-0041] Strauss, A. , & Corbin, J. (1990). Basics of qualitative research: Grounded theory procedures and techniques (2nd ed.). Newbury Park, CA: Sage.

[nop2374-bib-0042] Sveinsdóttir, H. , Ragnarsdóttir, E. D. , & Blöndal, K. (2016). Praise matters: The influence of nurse unit managers’ praise on nurses’ practice, work environment and job satisfaction: A questionnaire study. Journal of Advanced Nursing, 72, 558–568. 10.1111/jan.12849 26564786

[nop2374-bib-0043] Thériault, M. , Dubois, C.‐A. , Borgès da Silva, R. , & Prud’homme , A. (2019). Nurse staffing models in acute care: A descriptive study. Nursing Open, 6, 1218–1229. 10.1002/nop2.321 31367448 PMC6650648

[nop2374-bib-0044] Tong, A. , Sainsbury, P. , & Craig, J. (2007). Consolidated criteria for reporting qualitative research (COREQ): A 32‐item checklist for interviews and focus groups. International Journal for Quality in Health Care, 19, 349–357. 10.1093/intqhc/mzm042 17872937

[nop2374-bib-0045] Van Breugel, G. , Van Olffen, W. , & Olie, R. (2005). Temporary liaisons: The commitment of “temps” towards their agencies. Journal of Management Studies, 42, 539–566. 10.1111/j.1467-6486.2005.00508.x

[nop2374-bib-0046] Xue, Y. , Chappel, A. R. , Freund, D. A. , Aiken, L. H. , & Noyes, K. (2015). Cost outcomes of supplemental nurse staffing in a large medical center. Journal of Nursing Care Quality, 30, 130–137. 10.1097/ncq.0000000000000100 25479239

